# Gigantic rare left atrial appendage with four lobes closed using a watchman FLX and LAmbre closure device in the kissing technique: a case report

**DOI:** 10.1093/ehjcr/ytag383

**Published:** 2026-05-22

**Authors:** Abdelrahman Elhakim, Yannick Britt, Mohamed Elhakim, Osama Bisht

**Affiliations:** Cardiology Department, Heart Center Braunschweig, Salzdalumer St. 90, Braunschweig 38126, Germany; Department of Cardiology, Schoen Hospital Neustadt in Holstein, Am Kiebitzberg 10, 23730 Neustadt, Germany; Department of Cardiology, Schoen Hospital Neustadt in Holstein, Am Kiebitzberg 10, 23730 Neustadt, Germany; Intensive Care Medicine Department, The Royal Prince Alfred Hospital, 50 Missenden Rd, Camperdown, NSW 2050 Sydney, Australia; Interventional Cardiology Consultant, Heart Center Coswig, Lerchenfeld 1, Coswig (Anhalt) 06869, Germany

**Keywords:** Atrial fibrillation, Left atrial appendage closure, Oral anticoagulation, Wachman FLX occluder, LAmbra occluder, Case report

## Abstract

**Background:**

Despite the growing experience with left atrial appendage closure and the variety of closure devices and techniques, challenges remain in some cases due to anatomical complexities and varieties.

**Case summary:**

We report a case of rare and complex LAA anatomy. Cardiac computed tomography and angiography demonstrated a gigantic LAA with four lobes at the level of the ostium that cannot be occluded by the currently approved LAA closure devices in Europe. Watchman FLX (27 mm plug) and LAmbre (16/22 mm lobe/disc) closure devices in the kissing technique were successfully deployed under fluoroscopy and transoesophageal echocardiogram guidance without procedure-related complications.

**Discussion:**

Watchman FLX (plug) and LAmbre (umbrella and disc) Kissing is feasible. The LAmbre device may close very small or large LAAs with anatomical complexity.

Learning points

- Complex left atrial appendages are being more frequently encountered, so more innovations and pushing the limits with calculated risk are needed.
- The kissing technique for left atrial appendage occlusion is safe and feasible for LAA staged closure when necessary.
- This case provides a unique approach for left atrial appendage occlusion using the Watchman FLX and LAmbra devices.

## Introduction

Atrial fibrillation (AF) is the most prevalent cardiac arrhythmia. Its incidence is increasing with an estimated rate of2–4%.^1^

This population Group, the left atrial appendage (LAA), was identified as a primary source of thromboembolism. While oral anticoagulation (OAC) is the current standard of care, discontinuation over time is increasing due to major bleeding events, chronic renal failure, perceived high bleeding risk, drug side effects, and patient incompliance.

In recent decades, LAA closure (LAAC) has served as an alternative to reduce stroke and bleeding events in high-risk AF patients who cannot tolerate anticoagulation or have a contraindication.

LAAC is upgraded in the 2023 ACC/AHA/ACCP/HRS AF guidelines, moving from a Class 2b to a Class 2a indication for patients with a moderate-to-high risk of stroke and a contraindication to long-term OAC.^2^

In contrast, the 2024 ESC guideline for percutaneous LAAC indication still has a Class 2b recommendation.^3^

Due to a large variety and complexity of different LAA morphologies, there is still no ideal percutaneous LAAC device suitable for all anatomies. In addition, LAAC-related complications remain an Achilles’ heel, clinicians often face the dilemma of balancing the benefits and risks such as device-related thrombus (DRT), peri-device leak (PDL), device embolization or migration, pericardial effusion, air embolism, and compression of the coronary artery.^4^

The US Food and Drug Administration (FDA) approved catheter-based LAAC using the Watchman single seal (Boston Scientific, Marlborough, US) and the Amulet double seal devices (Abbott Vascular, IL, US).

LAmbra device (Lifetech Scientific Corp., Guangdong, China) is still in the pre-market clinical trial phase.

Surgical LAAC can be performed using operator- and technique-dependent complete closure success.^5^

However, more innovations are required to overcome the limitations of current designs and sizing requirements, the need for coaxial deployment, and the use of antithrombogenic coverings.

Post-procedural antithrombotic therapy is used to ensure endothelialization of the device surface and prevent DRT. Depending on the LAAC indications, the choice and duration of antithrombotic therapy should be individualized.^6^

We report a complex case of a rare and large LAA with four lobes that was successfully closed with two different LAAC devices.

## Summary figure

**Figure:**
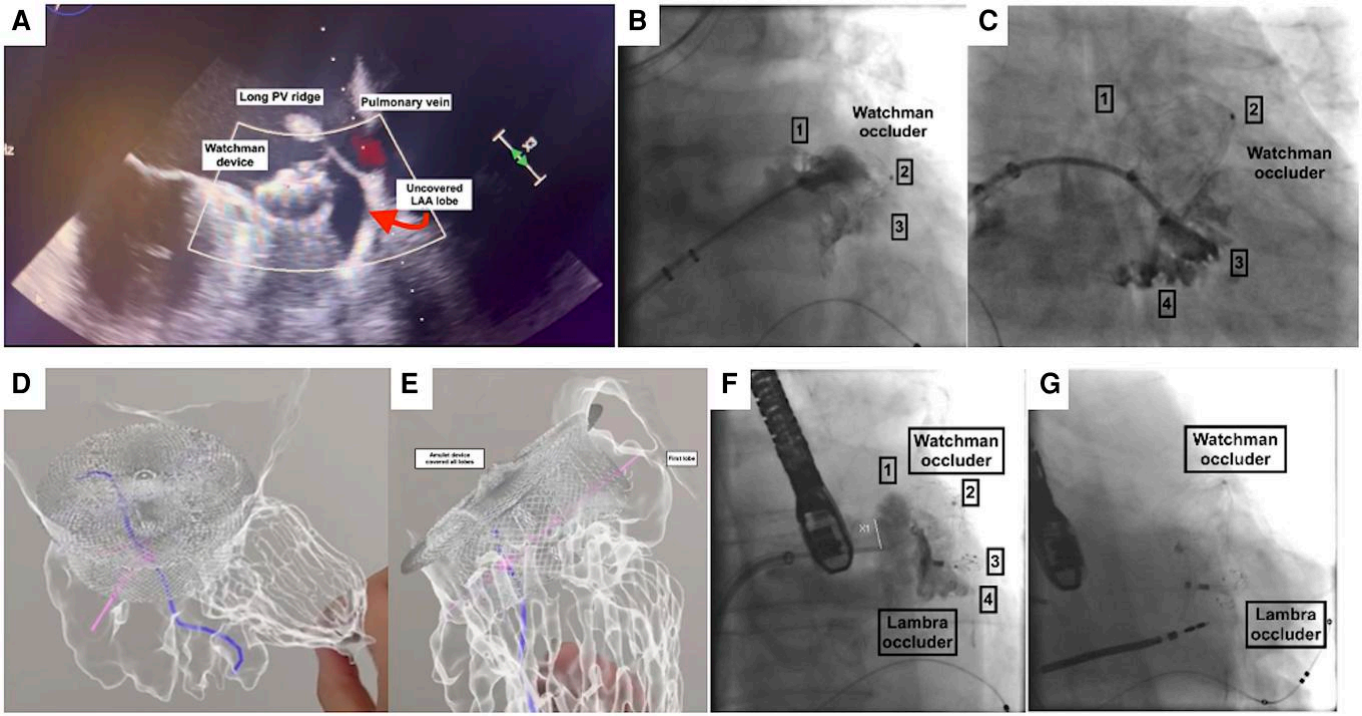


## Case presentation

An-86-year-old hypertensive man with known chronic kidney disease (creatinine 2 mg/dL and GFR 30 mL/min), previous implantation of CRT-D due to ischaemic cardiomyopathy, and EF ca. 35% and permanent AF in therapy with apixaban 2,5 mg bid. He suffered from microcytic anaemia, Hb 8 g/dL, and multiple petechial haemorrhages.

His CHA_2_DS_2_VA score was 5 and his HAS-BLED score was 3.

**Figure 1:**
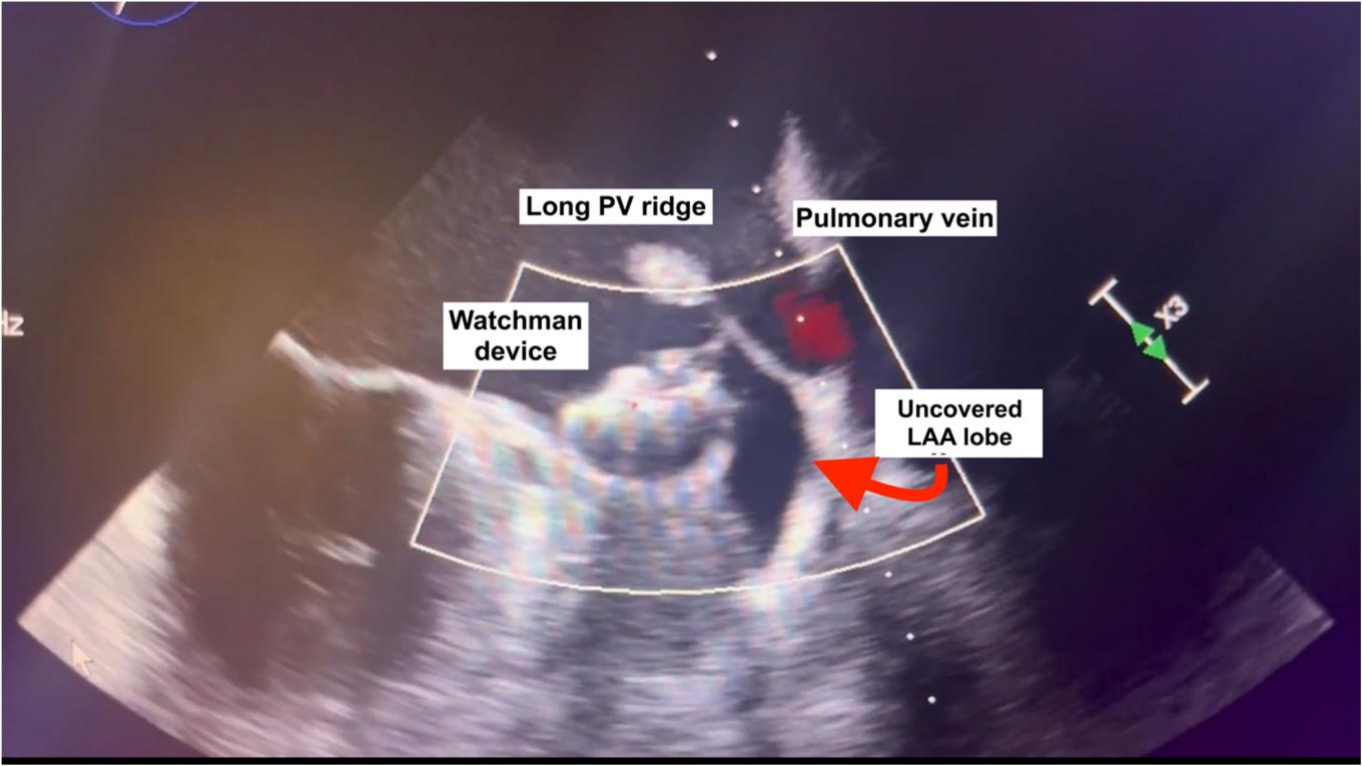
2D-TOE image during initial LAAC procedure showed the bilobed LAA configuration in the 90° view, the larger lobe was covered with watchman device, the smaller lobe was uncovered lobe and long pulmonary vein ridge. PV: pulmonary vein; LAAC: left atrial appendage closure.

**Figure 2:**
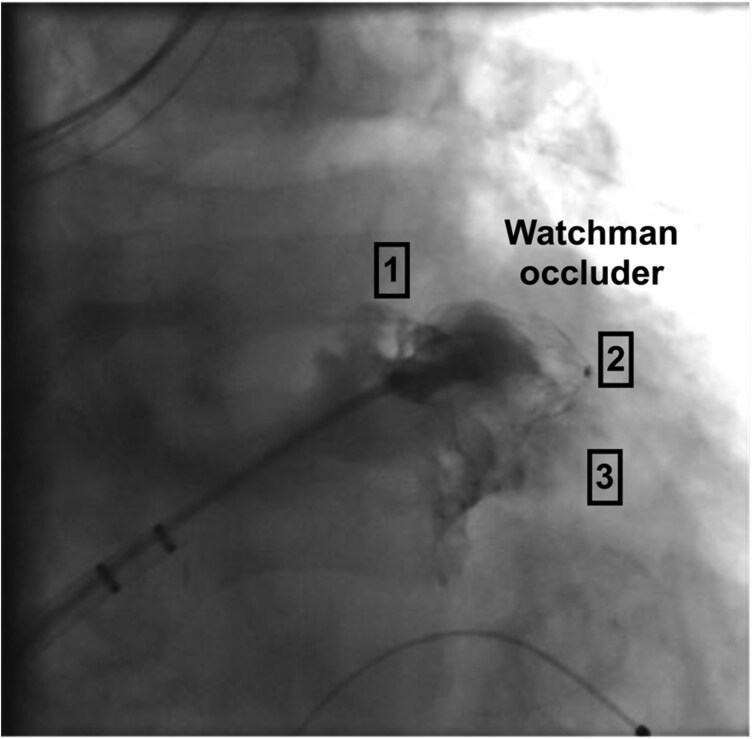
Fluoroscopy image of LAA in right caudal angulation of 20/20 showed an open small first lobe, second lobe with successful placement of a watchman device and an open third lobe. LAA: left atrial appendage.

**Figure 3:**
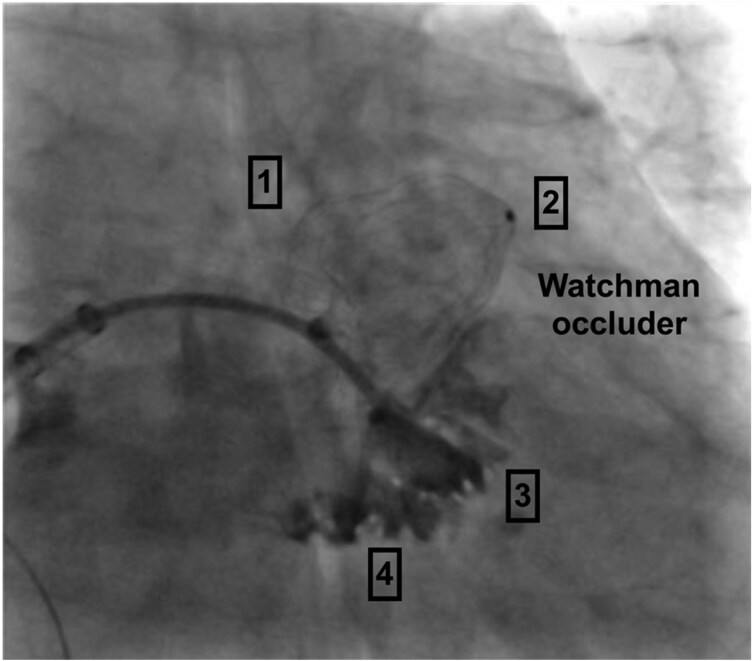
Fluoroscopy image of LAA in right caudal angulation of 20/20 showed the second lobe with successful placement of a watchman device and an open third and fourth lobes. Note the white line between the third and fourth lobes indicate the proximal bifurcation. LAA: left atrial appendage.

Therefore, he underwent LAAC with the implantation of a Watchman 27-mm device. After implantation, TOE (*Figure 1*) and angiography revealed uncovered first, third, and fourth lobes (*Figures 2* and *3*).

A cCT confirmed the crescent-shaped patent LAA ostium of 40 mm length, next to the implanted Watchman, the ostium of the first lobe was 6 mm width and 10 mm length, and 8 mm width ×16 mm length for the third and fourth lobes.

We decided to proceed with the occlusion of the third and fourth lobes. However, there was no device available for this size, so we decided to implant the 16/22 mm LAmbra smallest device with a larger disc that would simultaneously cover the third and fourth lobes for off-label use (instructions for use with minimal diameter 11 mm). Plan B with other devices was considered, including an Amplatzer Vascular Plug II (Abbott) as off-label use.

**Figure 4:**
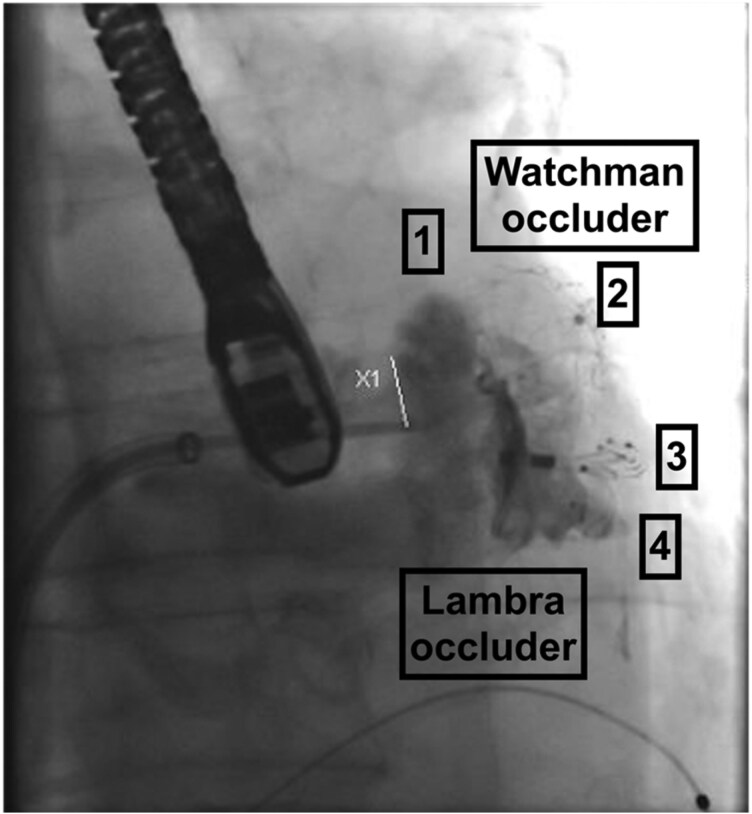
Final fluoroscopy image of LAA in right caudal angulation of 20/20 showed successful placement of watchman and LAmbra devices in the kissing technique with an open small first lobe. The LAmbra disc covered the third and fourth lobes. LAA: left atrial appendage.

The device was delivered using a 10-F steerable delivery sheath to the LAA. The procedure and handling of the device were very challenging. Selective umbrella deployment in the third lobe and finally disc deployment were able to seal the third and fourth lobes confirmed by intraoperative fluoroscopy and TOE with a small leak of 4 mm (*Figure 4*) (see Supplementary material online, *Videos S1*–S4).

The OAK was discontinued, and the patient was set on aspirin 100 mg and clopidogrel 75 mg daily for 3 months, followed by lifetime aspirin 100 mg. The patient was discharged without periinterventional complications.

Follow-up at 45 days, transoesophageal echocardiography (TOE) showed adequate sealing and a small PDL of 4 mm.

Regarding the first lobe, the size of 6 mm width as well as the conic shape with 10 mm depth cannot provide adequate anchoring with the currently available LAAC devices. We decided to let it patent due to patient willingness and comorbidities, and plan to close it with Vascular Plug if the patient developed DRT.

## Discussion

This is a rare case of LAA with four lobes. The challenges were different axes, limitations of current designs to close it, and the need for coaxial deployment.

In addition, commercially available LAAC devices allow the closure of the LAA ostium with a minimal diameter of 11 mm and a maximal diameter of 52 mm.

Moreover, incomplete LAA closure is associated with an increased risk of cerebral and systemic embolization.

Cardiac computed tomography (cCT) provides insights into challenging anatomical features, aiding in accurate device sizing and procedural planning.

In addition, two- and three-dimensional TOE and intracardiac echocardiography (ICE) provide dynamic and real-time imaging capabilities and help clinicians anticipate complications such as PDL or DRT.

Recently, the European Left Atrial Appendage Closure Club (ELAACC) developed a new classification for distinguishing between standard and complex LAAC cases and provided a quantitative assessment that aids in preprocedural planning and risk management to improve outcomes. It is based on five key LAA parameters: Entrance, landing zone, anatomy, axis and contraction.^7^

After LAAC of the second lobe, meticulous assessment of the LAA anatomy using multimodality images was the key for optimal management. The steerable delivery sheath was necessary to overcome the coaxial difficulties.

According to the ELAAC classification, this case had 11 points (5 points indicate highly complex anatomy) as follows:

**Entrance/ostium**: presence of a large ostium diameter of 40 mm, a long pulmonary vein ridge, and a low ostium position (3 points).

**Landing zone:** High ostial eccentricity. A proximal first, third and fourth lobes and proximal bifurcation between the second, third, and fourth lobes (4 points).

**Anatomy:** early sharp bend, short depth, and funnel-shaped first, third, and fourth lobes (3 points).

**Axis of the LAA:** Axis deviation of the first, third, and fourth lobes (1 point).

In multilobulated LAA, the authors recommend first trying to place the LAmbra device. It has a unique design of variable combinations of different sizes of the disc to adequately seal the LAA ostium. A custom-made device is also available on demand. Barocelli *et al*.^8^ reported a case of a giant windsock LAA successfully closed using a custom-made LAmbre device 38/46 mm.^5^

Moreover, the umbrella with its stabilizing hooks localized in the LAA body enables successful anchoring.

Another strategy is to implant the Watchman device followed by the Amulet or LAmbra device, with the disk overlapping, as illustrated in our case.

To the best of our knowledge, this is the first reported LAA case with four lobe anatomy being treated successfully with two different sealing mechanisms of LAAC devices without complications.

This case highlights the safety and feasibility of the staged closing of a quadrilobed LAA with different LAAO devices when needed. However, it warrants further prospective long-term safety and efficacy evaluation.

## Conclusion

Complex left atrial appendages are being more frequently encountered. The LAmbre device may be considered to close very small or large multilobulated LAA with anatomical complexity.

Watchman FLX and LAmbra devices in the kissing technique is safe and feasible for LAA staged closure when necessary.

## Lead author biography


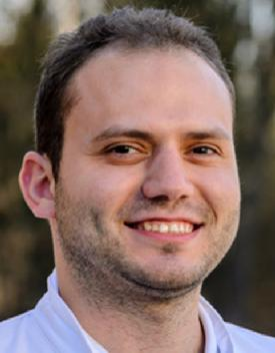
Interventional cardiologist, subspeciality in structural heart intervention and intensive medicine.

## Supplementary Material

ytag383_Supplementary_Data

## Data Availability

All data are incorporated into the article and its online Supplementary material. The data underlying this article are available in the article and in its online Supplementary material. The paper is not under consideration elsewhere; none of the paper's contents have been previously published; all authors have read and approved the manuscript.
